# The Sweetener-Sensing Mechanisms of the Ghrelin Cell

**DOI:** 10.3390/nu8120795

**Published:** 2016-12-07

**Authors:** Sandra Steensels, Laurien Vancleef, Inge Depoortere

**Affiliations:** Gut Peptide Lab, Translational Research Center for Gastrointestinal Disorders (TARGID), University of Leuven—KU Leuven, 3000 Leuven, Belgium; sandra.steensels@kuleuven.be (S.S.); laurien.vancleef@kuleuven.be (L.V.)

**Keywords:** ghrelin, sweet taste receptor, glucose, sweeteners, gustducin

## Abstract

Carbohydrate administration decreases plasma levels of the ‘hunger hormone’ ghrelin. The ghrelin cell is co-localized with the sweet taste receptor subunit, TAS1R3, and the gustatory G-protein, gustducin, both involved in the sensing of sweeteners by entero-endocrine cells. This study investigated the role of gustducin-mediated sweet taste receptor signaling on ghrelin secretion in a gastric ghrelinoma cell line, tissue segments and mice. The monosaccharide d-glucose and low-intensity sweetener oligofructose (OFS) decreased (*p* < 0.001) ghrelin secretion while the high-intensity sweetener sucralose increased (*p* < 0.001) ghrelin secretion in vitro. These effects were not mediated via the sweet taste receptor or glucose transporters (the sodium-dependent glucose cotransporter SGLT-1 and GLUT2). The effect of these compounds was mimicked ex vivo in gastric and jejunal segments from both wild type (WT) and α-gustducin knockout (α-gust^−/−^) mice. In vivo, the sensing of d-glucose was polarized since intragastric but not intravenous administration of d-glucose decreased (*p* < 0.05) ghrelin levels in an α-gustducin independent manner which involved inhibition of duodenal ghrelin release. In contrast, neither OFS nor sucralose affected ghrelin secretion in vivo. In conclusion, α-gustducin-mediated sweet taste receptor signaling does not play a functional role in the sensing of carbohydrates, or low- or high-intensity sweeteners by the ghrelin cell.

## 1. Introduction

Over the past four decades, we have transitioned from a world in which underweight prevalence was more than double that of obesity, to one in which more people are obese than underweight [1]. This increase in obesity prevalence has been linked to an excessive sugar intake [2,3]. Therefore guidelines in healthcare arose, recommending reductions in added sugar intake [4]. Sugar replacers, such as high-intensity sweeteners (HIS, e.g., sucralose), can help reduce the sugar content of meals without affecting its taste. These sweeteners are non-caloric but might not be metabolically inert, since contradictory results have been reported on their impact on energy homeostasis [5].

Next to these HIS, prebiotic sweeteners such as oligofructose (OFS) have been proposed as functional food ingredients. OFS has a low caloric content (1.7 kcal/g) which is derived from its microbial fermentation products, the short-chain fatty acids (SCFAs), which can be used as an energy substrate by the colonocytes. It has a sweetening power of 35% of that of sucrose (table sugar) [6]. Furthermore, OFS decreases food intake, fat mass development, and hepatic steatosis in normal and obese rodents [7,8,9,10,11,12,13]. In humans, contradictory results have been reported with inulin-type fructans on body weight reduction [14].

The hunger hormone ghrelin can stimulate food intake, prevent fat utilization, increase body weight, inhibit glucose-induced insulin release and stimulate gastrointestinal motility [15,16,17,18]. Ghrelin needs a post-translational modification, catalyzed by the enzyme ghrelin-*O*-acyltransferase (GOAT) to exert its biological activity [15,19,20]. Both GOAT and ghrelin are present in X/A like cells of the gastric oxyntic mucosa.

Plasma ghrelin levels increase before a meal and decrease thereafter to determine the frequency of the meals. Whereas the preprandial rise involves activation of the autonomic nervous system [21], the magnitude of the postprandial decline is dependent on the macronutrient composition of the meal [22]. Whether the latter is mediated via pre- or postabsorptive effects or involves chemosensation by the ghrelin cell is still not clear. However, recent evidence suggests that the ghrelin cell is equipped not only with receptors for neuropeptides but also with receptors for dietary and endogenous metabolites such as amino acids and free fatty acids that can directly regulate ghrelin release [23,24]. Immunohistochemical studies also provided evidence for the presence of gustatory G-proteins (gustducin, transducin) [25] and a subunit of the sweet taste receptor (TAS1R2-TAS1R3) [26] on the ghrelin cell but their functional role remains to be elucidated. The sweet taste receptor is broadly tuned to detect glucose and other simple sugars, and is activated by artificial sweeteners [27]. The sweet taste receptor, coupled to gustducin, and the sodium-dependent glucose cotransporter (SGLT-1) act as glucose-sensors of the L-cells in the small intestine [28,29].

This study aimed to investigate whether α-gustducin mediated sweet taste receptor signaling is involved in the effect of carbohydrates and sweeteners on ghrelin release. A ghrelinoma cell line was used to investigate the in vitro effect and the mechanism of action of carbohydrates and sweeteners (sucralose and OFS) on ghrelin release. Ex vivo gastric and jejunal segments from wild type (WT) and α-gustducin (α-gust^−/−^) mice were used to determine whether the sweet sensing mechanisms of the ghrelin cell are tissue dependent and involve a sweet taste receptor coupled to the gustatory G-protein, α-gustducin. Finally, the effect of glucose and sweeteners on ghrelin release was investigated in vivo in WT and α-gust^−/−^ mice to investigate the role of α-gustducin mediated sweet taste receptor activation and signaling.

## 2. Materials and Methods

### 2.1. Materials

d-glucose was obtained from Merck (Merck, Darmstadt, Germany), sucralose, phloridzin and phloretin were purchased from Sigma-Aldrich (Sigma-Aldrich, St. Louis, MO, USA). OFS was kindly provided by Beneo-Orafti (Beneo-Orafti, Mannheim, Germany) and gurmarin by Prof. L. Briand (Center for Taste and Feeding Behaviour, Dijon, France). The stock solutions of phloretin and phloridzin were made in dimethylsulfoxide (DMSO) and further diluted in Krebs-Ringer buffer with 11 mM d-glucose resulting in a final concentration of 0.001% DMSO for 10 µM phloretin/phloridzin and 0.002% DMSO for 20 µM phloretin,. The ghrelinoma cell line, MGN3-1, was kindly provided by Prof. H. Iwakura (Kyoto University Hospital, Kyoto, Japan).

### 2.2. Mice

Male C57BL/6 WT mice and α-gust^−/−^ mice (kindly provided by Prof. R. Margolskee, Monell Chemical Senses Center, Philadelphia, PA, USA) were kept in the animal facility. All mice were housed (20–22 °C) under a 14-h:10-h light-dark cycle and had ad libitum access to food and drinking water. All experimental procedures were approved by the Ethical Committee for Animal Experiments of the KU Leuven (P100/2013).

### 2.3. Experimental Design

Overnight-fasted mice were either gavaged (150 µL) with d-glucose (4 g/kg body weight), OFS (5.6 g/kg body weight), sucralose (8.95 mg/kg body weight) or 0.9% NaCl, or injected intravenously (IV, 150 µL) into the tail vene with 1 g/kg body weight d-glucose or 0.9% NaCl. In humans sucralose is in general 320–1000 times sweeter than sucrose and sucrose is 1.25–1.43 times more sweet than glucose [30,31]. This indicates that sucralose is about 1000 times sweeter compared to glucose, resulting in a dose of 8.95 mg/kg for sucralose compared to 4 g/kg for glucose. Furthermore, OFS is 2–3.3 times less sweet than sucrose [6], resulting in a dose of about 5.6 g/kg for OFS. These doses were chosen to be “equisweet” in order to study the effect of the sweeteners after a similar degree of sweet taste receptor activation. However, the “equisweet” doses were based on human studies, although the dose used in human studies does not necessarily apply to mice.

Forty minutes after IV injection or gavage, mice were humanely killed. Blood was collected by cardiac puncture and supplemented with 4-(2-aminoethyl)benzenesulfonyl fluoride hydrochloride (4 mM) and ethylenediaminetetraacetic acid (1 mg/mL). Plasma was acidified (0.1 N HCl) and stored at −80 °C. The stomach and duodenum were removed and stored for protein extraction.

### 2.4. Ghrelin Tissue Extraction

Tissue from stomach and duodenum was boiled for 10 min followed by homogenization in three volumes of water with protease inhibitors (MP Biomedicals, Santa Ana, CA, USA) and nine volumes of 6% acetic acid. After 10 min of boiling, the homogenate was centrifuged to collect the supernatant which was diluted and subjected to radioimmunoassay (RIA). Protein levels were determined using the Pierce BCA Protein Assay Kit (Thermo Fisher Scientific Inc., Waltham, MA, USA).

### 2.5. Ghrelin Release from Intestinal Segments

Overnight fasted WT and α-gust^−/−^ mice were refed for two hours prior to being sacrificed. Segments of the intact corpus of the stomach (0.3 × 0.3 cm) and jejunum (0.4 × 1 cm) were dissected and incubated at 37 °C in Krebs-Ringer buffer (11 mM d-glucose) with the test solutions (d-glucose (200 mM), OFS (10%), sucralose (200 mM)) for 2 h. The culture medium was collected, acidified (0.1 N HCl) and stored at −80 °C. Tissue segments were dried to correct ghrelin release for dry tissue weight of the segment.

### 2.6. Ghrelin Release from Ghrelinoma Cells

MGN3-1 cells were cultured in Dulbecco’s modified eagle medium (DMEM, Sigma Aldrich) supplemented with 10% fetal bovine serum (FBS) and 1% penicillin and streptomycin. Cells were incubated with d-glucose (11.1–200 mM), d-fructose (20–200 mM), OFS (0.1%–10%) or sucralose (1–200 mM) in Krebs-Ringer buffer with 11 mM d-glucose for three hours. Osmolality was corrected to physiological levels by adapting the concentration of NaCl. The effect of the sweet taste receptor antagonist (30 µg/mL gurmarin) [32], or glucose transporter inhibitors (SGLT1 antagonist; 10 µM phloridzin [33], glucose transporter (GLUT) family antagonist; 10–20 µM phloretin [34]) was investigated by preincubation of the cells for 30 min with the respective inhibitors after which the culture medium was removed and replaced by a combination of the antagonist and the indicated carbohydrate or sweetener for three hours. The dose of gurmarin was high enough to block both TAS1R2-TAS1R3 [32] and the TAS1R3 homodimer since this dose blocked the umami taste receptor (TAS1R1-TAS1R3) and thus the common subunit of the sweet and umami taste receptor, namely TAS1R3 [23,35]. Following the incubation, the supernatant was collected, acidified (0.1 N HCl) and stored at −80 °C.

### 2.7. Radioimmunoassay (RIA)

Plasma samples and cell/tissue culture supernatants were extracted on a SEP-Pak C18 cartridge (Waters Corporation, Milford, MA, USA), vacuum-dried and subjected to ghrelin RIA as previously described [25]. For determination of octanoyl ghrelin a rabbit anti-human ghrelin [1,2,3,4,5,6,7,8] antibody was used which does not recognize desoctanoyl ghrelin. Total ghrelin levels were determined using a rabbit anti-human ghrelin [14,15,16,17,18,19,20,21,22,23,24,25,26,27,28] antibody, which recognizes both octanoyl and desoctanoyl ghrelin.

### 2.8. Quantitative Real-Time PCR (qRT-PCR)

Total RNA was isolated from MGN3-1 cells and tissue segments from the mouse gastro-intestinal (GI) tract using the RNeasy kit (Qiagen, Antwerp, Belgium), treated with Turbo DNAfree kit (Ambion, Carlsbad, CA, USA) and reverse transcribed to cDNA using Superscript II Reverse Transcriptase (Invitrogen, Carlsbad, CA, USA). The qRT-PCR reaction was performed as described previously, using the Lightcycler 480 (Roche Diagnostics, Brussels, Belgium) with the Lightcycler 480 Sybr Green I Master mix (Roche Diagnostics, Brussels, Belgium) [36], and analyzed using the LightCycler^®^ 480 SW 1.5.1 software (Roche Diagnostics, Brussels, Belgium). Results were expressed relative to glyceraldehyde 3-phosphate dehydrogenase (GAPDH). The following primers were used: GAPDH: forward CCCCAATgTgTCCgTCgTg, reverse gCCTgCTTCACCACCTTCT; SGLT-1: forward CggAAgAAggCATCTgAgAA, reverse AATCAgCACgAggATgAACA; GLUT2: forward TCTTCACggCTgTCTCTgTg, reverse AATCATCCCggTTAggAACA; TAS1R2: forward gCACCAAgCAAATCgTCTATCC, reverse ATTgCTAATgTAggTCAgCCTCgTC; TAS1R3: forward CAggCAgTTgTgACTCTgTTg, reverse TgCgATgCAgATACCTCgTg.

### 2.9. Statistical Analysis

The data representing the effect of the test compounds on ghrelin release from intestinal segments and on plasma ghrelin levels and tissue ghrelin content were assessed for normality of distribution. As the data were distributed in a non-normal and/or non-homogeneous manner, log-transformed data were used to examine the main effects of the compounds using multivariate analysis of variance (MANOVA). An interaction effect between compounds and genotypes was included in the model as well. Post-hoc *t*-tests with Holm-Sidak correction for multiple testing were applied (SAS Studio University Edition 9.4). Results are presented as predicted values ± standard error of the predicted values.

Dose-response curves of the test compounds in the MGN3-1 cell line are represented as ± standard error of the mean (SEM) and were analyzed using a repeated measures analysis (factors; compound and dose), followed by planned comparisons post-hoc testing and Bonferroni correction (Statistica 12, Statsoft). The effect of the different antagonists on the effect of the test compounds on ghrelin release was analyzed with two-way ANOVA, followed by planned comparisons post-hoc testing and Bonferroni correction (factors; compound and antagonists) (Statistica 12, Statsoft). Significance was accepted at the 5% level.

## 3. Results

### 3.1. In Vitro Studies in the MGN3-1 Ghrelinoma Cell Line

**Figure 1:**
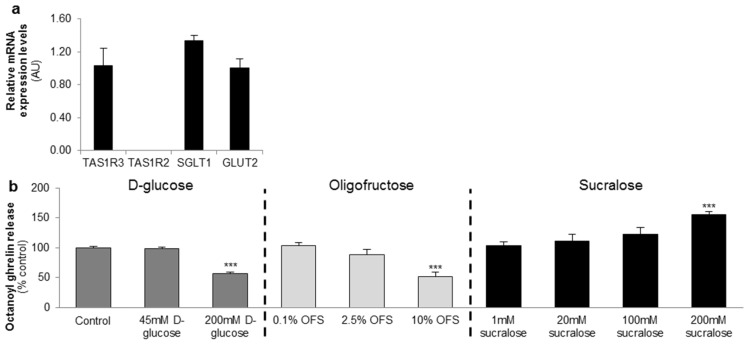
d-glucose and the low-intensity sweetener oligofructose (OFS) decrease octanoyl ghrelin release while a high-intensity sweetener sucralose increases octanoyl ghrelin secretion from a ghrelinoma cell line. (**a**) Relative mRNA expression levels of the two subunits of the sweet taste receptor (TAS1R2-TAS1R3), and the glucose (sodium-dependent glucose cotransporter (SGLT-1), glucose transporter 2 (GLUT2)) transporters in the ghrelinoma cell line, MGN3-1 (*n* = 3/sensor); (**b**) Concentration-dependent effect of 3-h stimulation with d-glucose, OFS and sucralose on octanoyl ghrelin release (*n* = 9–12). Results (mean ± standard error of the mean (SEM)) are expressed relative to the control stimulation (Krebs buffer containing 11.1 mM d-glucose). *** *p* < 0.001 vs. control. AU: arbitrary units.

The gastric MGN3-1 cell line shows a strong expression of the TAS1R3 subunit of the sweet taste receptor and the glucose transporters (SGLT1 and GLUT2). The sweet taste receptor subunit TAS1R2 is not detectable in the cell line (Figure 1a).

MGN3-1 cells were incubated with increasing concentrations of d-glucose, OFS or sucralose and the effect on octanoyl ghrelin release was determined.

d-glucose (200 mM) and OFS (10%) induced a significant (*p* < 0.001) decrease in octanoyl ghrelin levels while sucralose (200 mM) stimulated (*p* < 0.001) octanoyl ghrelin release. Lower concentrations had no effect (Figure 1b).

**Figure 2:**
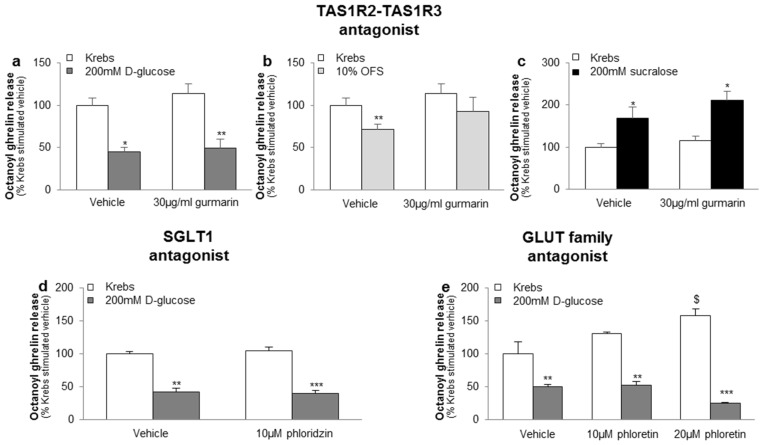
The effect of carbohydrates and sweeteners on octanoyl ghrelin release in the ghrelinoma cell line are not mediated via the sweet taste receptor or the glucose transporters. Effect of preincubation (30 min) of MGN3-1 cells with a (**a**–**c**) TAS1R2-TAS1R3 antagonist (gurmarin, 30 µg/mL, *n* = 9); (**d**) SGLT1 inhibitor (phloridzin, 10 µM, *n* = 9) or (**e**) GLUT family antagonist (phloretin, 10–20 µM, *n* = 9) or their respective vehicle (Krebs with or without dimethylsulfoxide (DMSO)) on the effect of (**a**,**d**,**e**) 200 mM d-glucose; (**b**) 10% OFS; and (**c**) 200 mM sucralose compared to Krebs buffer on octanoyl ghrelin release in MGN3-1 cells. Results (mean ± SEM) are expressed relative to the control stimulation (Krebs buffer containing 11.1 mM d-glucose). * *p* < 0.05, ** *p* < 0.01, *** *p* < 0.001 vs. vehicle, $ *p* < 0.05 vs. vehicle stimulated control.

The inhibitory effect of 200 mM d-glucose or 10% OFS and the stimulatory effect of 200 mM sucralose on octanoyl ghrelin release was not blocked by the sweet taste receptor antagonist gurmarin (30 µg/mL), the SGLT1 inhibitor phloridzin (10 µM) or the GLUT family inhibitor phloretin (10–20 µM) (Figure 2a–e). Phloretin (20 µM), but not phloretin (10 µM), phloridzin (10 µM) or gurmarin (30 µg/mL), increased basal ghrelin release with about 60% (*p* < 0.05) (Figure 2e).

### 3.2. Ex Vivo Studies in Intestinal Segments

**Figure 3:**
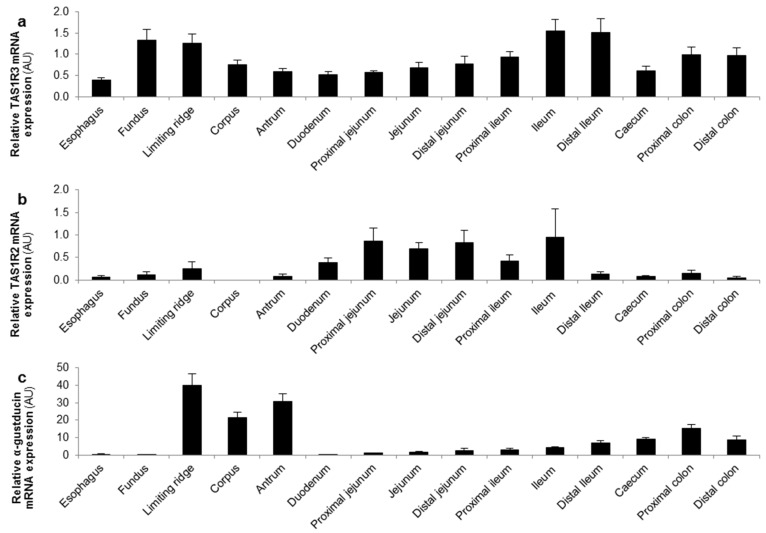

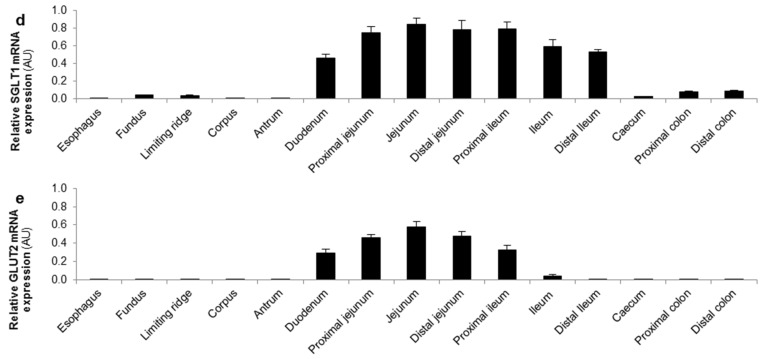
TAS1R3 and α-gustducin are expressed throughout the gastro-intestinal (GI) tract while TAS1R2 and the glucose transporters are expressed in the small intestine. Relative mRNA expression levels of (**a**,**b**) the two subunits of the sweet taste receptor (TAS1R2-TAS1R3); (**c**) α-gustducin and (**d**,**e**) the glucose (SGLT-1, GLUT2) transporters throughout the mouse GI tract (*n* = 5). Results are presented as mean ± SEM. AU: arbitrary units.

The mRNA expression levels of the different glucose sensors were determined in several regions of the GI tract of mice. The TAS1R3 subunit and α-gustducin were expressed throughout the GI tact with a high expression in the stomach and distal GI tract (Figure 3a,c). In contrast, the highest expression levels of the TAS1R2 subunit and the glucose transporters (SGLT-1 and GLUT2) were observed in the small intestine (Figure 3b,d,e).

The differential expression of the TAS1R3 subunit (corpus and jejunum) and the TAS1R2 subunit (only jejunum) allowed us to investigate, in the respective ex vivo segments, whether the effect of glucose and the high- and low-intensity sweeteners on ghrelin release is region-dependent and thus involves the TAS1R2-TAS1R3 receptor heterodimer. Furthermore, the effect of the compounds was tested in segments from WT and α-gust^−/−^ mice to elicit the role of the G-protein, α-gustducin, coupled to the sweet taste receptor complex.

#### 3.2.1. d-Glucose Decreased Ghrelin Release from Gastric and Jejunal Segments in an α-Gustducin Independent Manner

**Figure 4:**
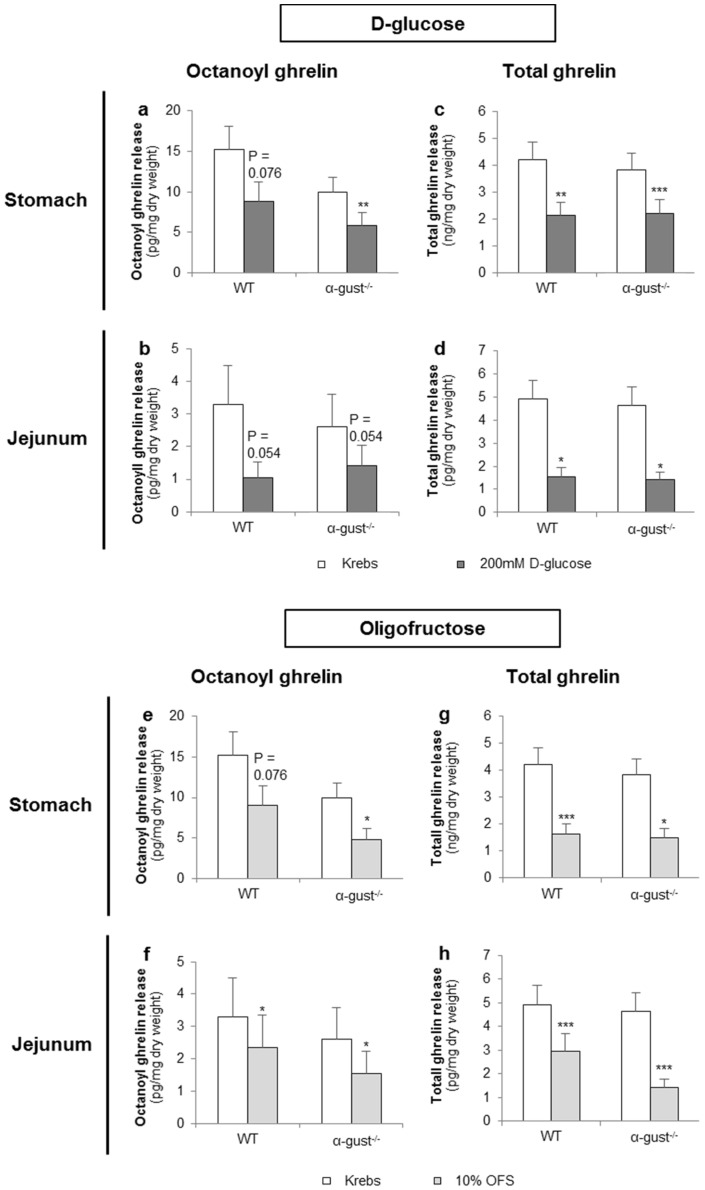
The effect of d-glucose and OFS on ghrelin release from segments of the corpus and jejunum is α-gustducin independent. Effect of 2-h stimulation with Krebs buffer or 200 mM d-glucose (**a**–**d**) or 10% OFS (**e**–**h**) on octanoyl (**a**,**b**,**e**,**f**) and total ghrelin release (**c**,**d**,**g**,**h**) from tissue segments of the corpus of the stomach (*n* = 6) or the jejunum (*n* = 6) from wild type (WT) and α-gustducin knockout (α-gust^−/−^) mice. Results are presented as predicted values ± standard error of the predicted values. * *p* < 0.05, ** *p* < 0.01, *** *p* < 0.001 vs. Krebs treated segments.

d-glucose (200 mM) tended to decrease octanoyl ghrelin release (WT: *p* = 0.076) from segments of the corpus of WT mice and decreased (α-gust^−/−^: *p* < 0.01) octanoyl ghrelin secretion from segments of the corpus of α-gust^−/−^ mice (Figure 4a). A similar effect was observed in jejunal segments from both WT and α-gust^−/−^ mice (Figure 4b). Accordingly, d-glucose decreased total ghrelin release from segments of the corpus (WT; *p* < 0.01, α-gust^−/−^; *p* < 0.001) and jejunum (WT; *p* < 0.05, α-gust^−/−^; *p* < 0.05) (Figure 4c,d). No interaction effects (genotype × treatment) were observed.

#### 3.2.2. OFS Decreased Ghrelin Release from Gastric and Jejunal Segments in an α-Gustducin Independent Manner

OFS (10%) tended to decrease (WT: *p* = 0.076) octanoyl ghrelin release from segments of the corpus in WT mice and decreased (α-gust^−/−^: *p* < 0.05) octanoyl ghrelin secretion from segments of the corpus in α-gust^−/−^ mice (Figure 4e). A significant (*p* < 0.05) OFS-induced reduction in octanoyl ghrelin release was also observed in jejunal segments (Figure 4f). Accordingly, OFS significantly decreased total ghrelin release from segments of the corpus and jejunum from both genotypes (Figure 4g,h). No interaction effects (genotype × treatment) were observed.

#### 3.2.3. Sucralose Increased Octanoyl Ghrelin, but Not Total Ghrelin Release from Gastric and Jejunal Segments in an α-Gustducin Independent Manner

**Figure 5:**
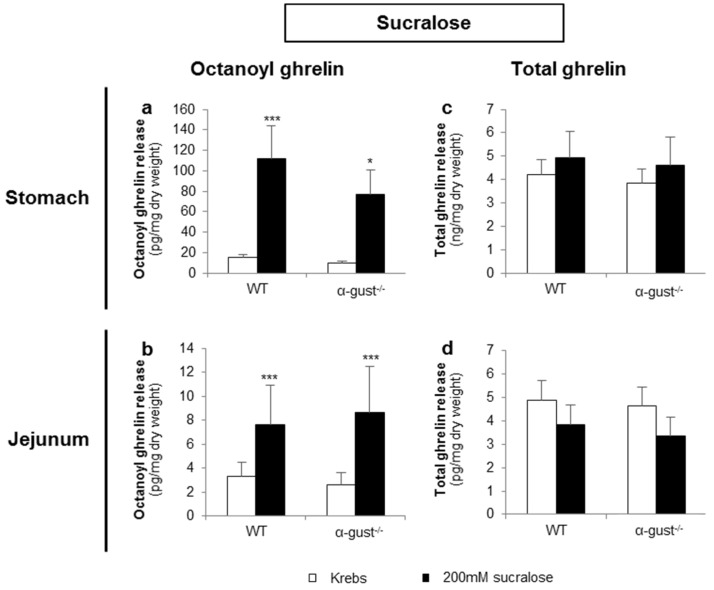
Sucralose increased octanoyl ghrelin, but not total ghrelin release from gastric and jejunal segments in an α-gustducin-independent manner. Effect of 2-h stimulation with 200 mM sucralose or Krebs buffer on (**a**,**b**) octanoyl and (**c**,**d**) total ghrelin release from tissue segments of the corpus of the stomach (*n* = 6) or the jejunum (*n* = 6) from WT) and α-gust^−/−^ mice. Results are presented as predicted values ± standard error of the predicted values. * *p* < 0.05, *** *p* < 0.001 vs. Krebs treated segments.

Sucralose (200 mM) significantly increased octanoyl ghrelin release from segments of the corpus and jejunum of both genotypes (Figure 5a,b). However, sucralose did not affect total ghrelin release from segments of either the corpus or jejunum in both genotypes (Figure 5c,d). No interaction effects were observed.

### 3.3. In Vivo Studies in Mice

The effect of the caloric and non-caloric sweeteners was tested in vivo in WT and α-gust^−/−^ mice to investigate the physiological role of sweet taste receptor activation.

#### 3.3.1. The Sensing of d-Glucose by the Ghrelin Cell Is Polarized and Occurs via the Lumen

**Figure 6:**
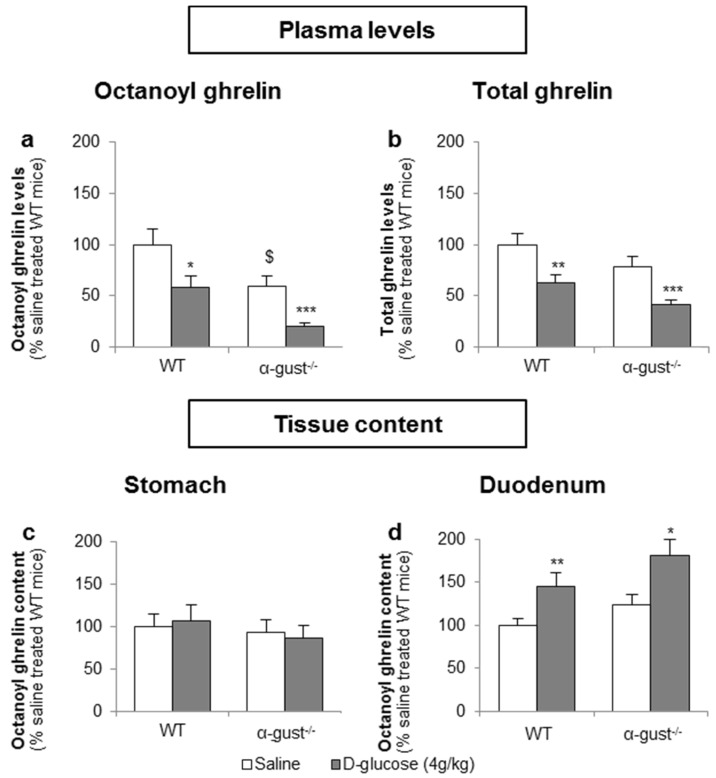
The inhibition of plasma ghrelin levels after an intragastric administration of d-glucose originates mainly from duodenal ghrelin cells. WT and α-gust^−/−^ mice were gavaged with d-glucose (4 g/kg, *n* = 8) or saline (*n* = 13). Ghrelin levels were determined in (**a**,**b**) plasma, (**c**) stomach and (**d**) duodenum, 40 min after administration. Results (predicted values ± standard error of the predicted values) are expressed relative to the control stimulation (saline treated WT mice). * *p* < 0.05, ** *p* < 0.01 and *** *p* < 0.001 vs. saline, $ *p* < 0.05 vs. saline-treated WT mice.

Basal fasted octanoyl ghrelin levels were 41% lower (*p* < 0.05) in α-gust^−/−^ control mice, compared to WT control mice. Intragastric administration of d-glucose (4 g/kg) in fasted mice significantly decreased plasma octanoyl (WT: −41% ± 11%; α-gust^−/−^: −48% ± 5%) and total ghrelin levels (WT: −38% ± 8%; α-gust^−/−^: −48% ± 4%) in both genotypes compared to vehicle-treated mice (Figure 6a,b). This was accompanied by an increase in duodenal tissue octanoyl ghrelin content in both WT (*p* < 0.01) and α-gust^−/−^ mice (*p* < 0.05) but not in gastric tissue octanoyl ghrelin content (Figure 6c,d).

**Figure 7:**
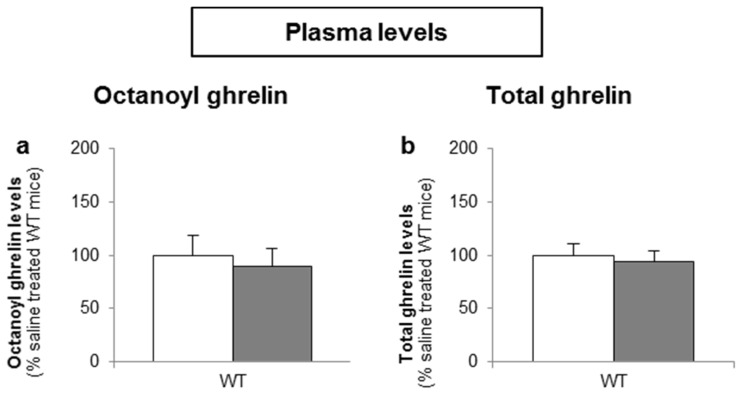

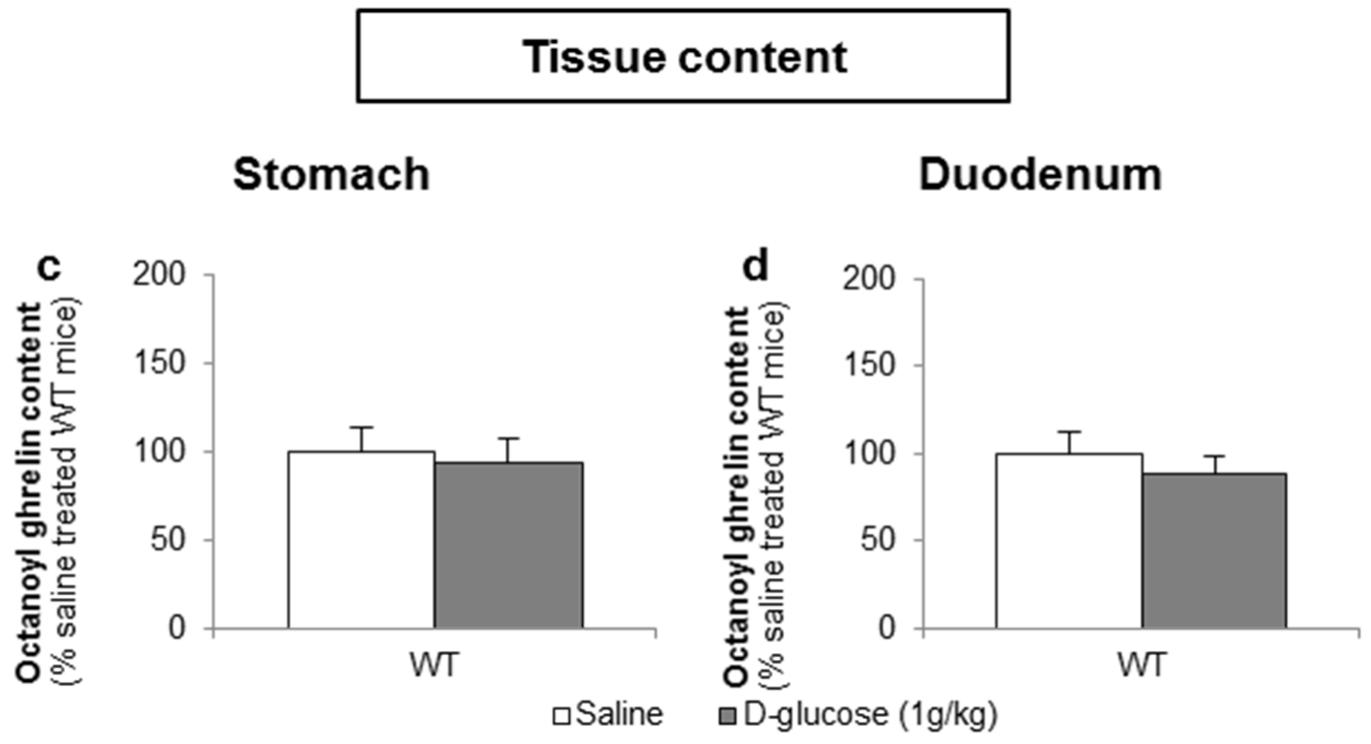
The sensing of d-glucose is polarized and occurs at the luminal side. Mice were intravenously injected with d-glucose (1 g/kg, *n* = 10) or saline (*n* = 11). Ghrelin levels were determined in (**a**,**b**) plasma, and in protein extracts from the (**c**) stomach and (**d**) duodenum, 40 min after administration. Results (predicted values ± standard error of the predicted values) are expressed relative to the control stimulation (saline treated WT mice).

To determine whether d-glucose is sensed via the lumen or the bloodstream after glucose uptake, the effect of intragastric (4 g/kg) versus intravenous administration of d-glucose (1 g/kg) on ghrelin release was compared. A pilot experiment showed that 1 g/kg d-glucose induced comparable peak blood glucose levels (330 ± 15 mg/dL) compared to the intragastric administration of 4 g/kg d-glucose (317 ± 31 mg/dL). Intravenous administration of d-glucose neither affected plasma octanoyl or total ghrelin levels nor gastric or duodenal tissue octanoyl ghrelin content (Figure 7a–d).

#### 3.3.2. Intragastric Administration of Neither a Low- nor a High-Potency Sweetener Affected Plasma Ghrelin Levels

**Figure 8:**
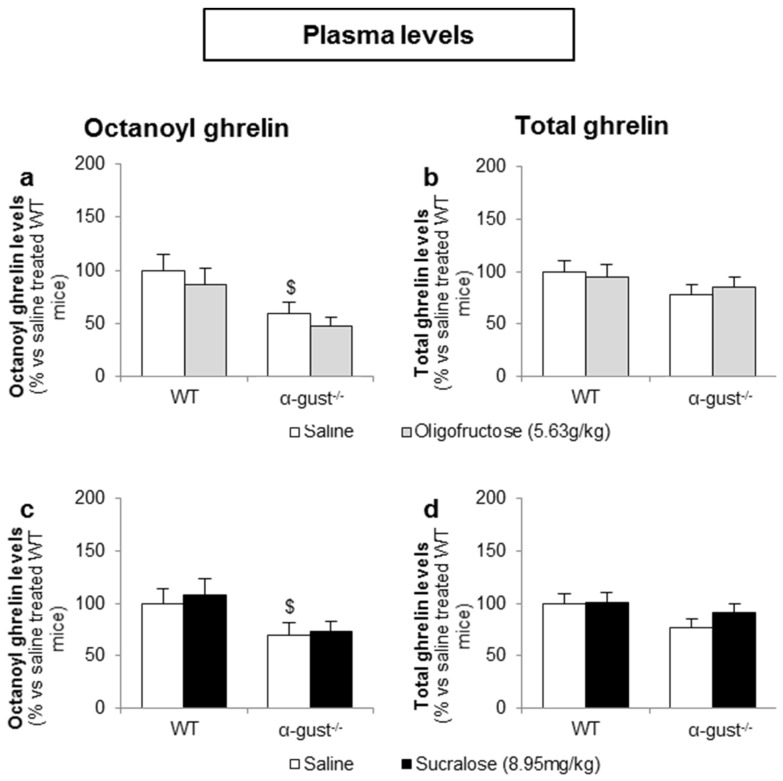
Intragastric administration of sweeteners does not affect ghrelin release. WT and α-gust^−/−^ mice were gavaged (**a**,**b**) OFS (5.63 g/kg) (*n* = 8), (**c**,**d**) sucralose (8.95 mg/kg) (*n* = 9) or saline (*n* = 9–13). Plasma octanoyl and total ghrelin levels were determined 40 min after administration. Results (predicted values ± standard error of the predicted values) are expressed relative to the control stimulation (saline treated WT mice). $ *p* < 0.05 vs. vehicle treated WT mice.

In contrast to d-glucose, intragastric administration of OFS (5.6 g/kg) or sucralose (9.0 mg/kg) did not affect plasma octanoyl or total ghrelin levels in either genotype (Figure 8a–d).

## 4. Discussion

The in vitro results in the ghrelinoma cell line and ex vivo results obtained in tissue segments showed that d-glucose and oligofructose (OFS) decreased ghrelin release at concentrations physiological to the postprandial luminal fluid. In contrast, the artificial sweetener sucralose increased ghrelin release at the supraphysiological concentration of 200 mM. Furthermore, neither α-gustducin mediated sweet taste receptor signaling nor glucose transport (SGLT-1, GLUT family) played a role in the effect of d-glucose, OFS or sucralose on ghrelin release.

Our in vivo findings indicate that the sensing of d-glucose by the X/A cells is polarized and occurred at the luminal side. The glucose-induced reduction in plasma ghrelin levels is α-gustducin-independent and originates from a reduced ghrelin release from duodenal, but not gastric cells. In contrast, the low-and high-intensity sweeteners (OFS and sucralose) did not elicit any changes in plasma ghrelin levels.

Previous studies showed that concentrations of d-glucose physiological to the postprandial basolateral concentrations were able to elicit changes in ghrelin secretion.

For instance, Sakata et al. showed that, compared to a low glycemic state (1 mM), normoglycemic (5 mM) and high (10 mM) concentrations of d-glucose decreased octanoyl ghrelin secretion from primary cultures of gastric mucosal cells [37]. Oya et al. showed that low (1 mM), normoglycemic (5 mM) and high (10 mM) concentrations of d-glucose increased ghrelin secretion compared to 25 mM d-glucose in MGN3-1 cells [38].

We could not confirm these findings and only observed an inhibition of ghrelin secretion at 200 mM d-glucose both in vitro and ex vivo. This dose mimics luminal glucose concentrations which range between 50 and 500 mM [39]. A similar observation was made for OFS which is usually supplemented at a dose of 8–21 g/day in the diet or in a drink, and results in 5%–10% OFS in the luminal fluid [14]. These findings suggest that the sensing of d-glucose and OFS may occur at the luminal side of the intestinal epithelium. This was confirmed by our in vivo studies which showed that intravenous administration of d-glucose did not affect plasma ghrelin levels. Immunohistochemistry studies previously showed that in contrast to “closed-type “ghrelin cells, which are not in contact with the lumen, “open-type” ghrelin cells show the presence of the TAS1R3-subunit in their apical cell pole contacting the lumen [26]. Since sweet taste receptors on the tongue are typically activated by 30–1000 mM glucose [40,41], the apical localization of the TAS1R3 subunit could explain the luminal sensing of d-glucose. Nevertheless, many open-type duodenal ghrelin cells also showed TAS1R3 staining in their basolateral domain. We have previously shown that in contrast to glucose, the sensing of amino acids is not polarized [23]. Since TAS1R3 is also involved in amino acid sensing, it is likely that the TAS1R3 staining in the basolateral domain is selectively involved in amino acid sensing.

The amount of sucralose in sweetened soft drinks represents about 0.4 mM and sucralose typically activates the sweet taste receptor at low millimolar concentrations [40]. Our findings therefore indicate that sucralose only stimulated ghrelin release at supraphysiological concentrations (200 mM).

The effect of sucralose on ghrelin release in the ghrelinoma cell line and ex vivo segments was opposite to those of d-glucose and OFS. Functional studies of the sweet taste receptor have revealed at least four binding sites for sweet-tasting compounds [42]. It is likely that low-intensity sweeteners (glucose and OFS) and high-intensity sweeteners such as sucralose will bind to a different binding site, possibly activating a different signaling cascade. Sugars are thought to increase cyclic adenosine monophosphate (cAMP) levels while artificial sweeteners may act by increasing levels of inositol trisphosphate (IP3) [43]. Still, both the cAMP and IP3 cascades eventually result in increased Ca^2+^ levels in the cell [43] and cannot explain why glucose and OFS decrease ghrelin release and sucralose stimulates ghrelin release.

Sucralose (>0.62 mM) has a bitter taste quality in rats [44], but not in humans [45]. However, it cannot be excluded that at high concentrations (200 mM) sucralose might also taste bitter in mice and humans. Activation of bitter taste receptors has been shown to stimulate ghrelin secretion in vivo in mice, partially via α-gustducin [25]. Since sucralose stimulated ghrelin secretion in segments from both WT and α-gust^−/−^ mice, it unlikely that the effect of sucralose is mediated via bitter taste receptors. Sucralose specifically increased octanoyl, but not total and thus desoctanoyl ghrelin release, therefore it might exert its activity through modulation of the activity of ghrelin-*O*-acyl transferase (GOAT). No evidence in literature so far supports the hypothesis of a link between sucralose and GOAT activity.

We could not assign an important role of the TAS1R2-TAS1R3 heterodimer in the effect of glucose and sweeteners on gastric ghrelin release. Indeed, the mRNA expression of the TAS1R2 subunit was absent in segments from the corpus and in the ghrelinoma cell line, which is of gastric origin. Other studies using the TAS1R2-lacZ knock-in mouse did not observe TAS1R2 expression in the stomach [46]. In contrast, Koyama et al. showed a very low expression of TAS1R2 in the MGN3-1 cell line and primary gastric ghrelin cells using RNA sequencing [47]. However, TAS1R3 may also function as a homodimer, as previously shown on the tongue [48], in adipocytes [49] and in pancreatic β-cells [50]. Zhao et al. showed that the TAS1R3 homodimer was not able to detect sweeteners and carbohydrates at low concentrations (<300 mM) [49]. This may explain why only high concentrations of glucose (200 mM), sucralose (200 mM) and OFS (10%) affected ghrelin secretion in the MGN3-1 cells.

However, the lack of effect of the sweet taste receptor antagonist, gurmarin, which has been shown to block the TAS1R3 subunit [23,35], on glucose and sweetener induced ghrelin release suggests that neither TAS1R2-TAS1R3 nor the TAS1R3 homodimer is important. Furthermore, the effect of d-glucose and the sweeteners did not differ between segments from WT and α-gust^−/−^ mice, indicating that gustducin-mediated signaling does not play an important role. However, it cannot be excluded that the sweet taste receptor heterodimer or the TAS1R3 homodimer can couple to other G-proteins than α-gustducin. Indeed, α-gust^−/−^ mice are not completely unresponsive to sweet compounds [51] and the TAS1R3 homodimer has been shown to couple to Gs in adipocytes [49]. Furthermore, indirect effects, mediated via glucose-induced GLP-1 release, seem unlikely since the effect should be blunted in segments from α-gust^−/−^ mice [28].

In L-cells, both α–gustducin mediated sweet taste receptor signaling and the glucose transporter, SGLT1, mediate glucose-induced GLP-1 secretion [28,29]. However, inhibitors for SGLT1 and the GLUT family could not confirm an involvement of these proposed glucose-sensors in the effect of d-glucose on octanoyl ghrelin secretion. Previous studies also suggested that ATP-sensitive potassium (K_ATP_) channels are involved in the effect of 25 mM d-glucose on ghrelin secretion in MGN3-1 cells [38]. However, tolbutamide (a potassium channel blocker) and diazoxide (a potassium channel activator) neither enhanced nor inhibited 1, 5 or 10 mM glucose-induced ghrelin secretion in primary cultures of gastric mucosal cells [37].

Our in vivo studies showed that the glucose-induced ghrelin inhibition was due to a tissue-specific inhibition of octanoyl ghrelin release from the duodenum. Williams et al. showed that intragastric infusion of glucose or water inhibited ghrelin release when gastric emptying was permitted but not when emptying was prevented, indicating that gastric chemosensation is not a sufficient trigger for ghrelin response [52]. Thus although our in vitro and ex vivo studies indicate that glucose can inhibit ghrelin secretion in the stomach, in vivo this glucose sensing may be ineffective.

Parker et al. showed that an intraduodenal glucose infusion proved to be just as effective in suppressing ghrelin levels as an intragastric infusion in healthy older men and women [53]. The magnitude of the glucose-induced decrease in plasma ghrelin levels was even dependent on the length of the small intestine exposed [54]. Tamboli et al. showed that jejunal glucose administration suppressed ghrelin levels to a greater degree compared with an intagrastric glucose administration in obese subjects. This was independent of circulation glucose levels, indicating that a nutrient-initiated signal in the jejunum may have regulated ghrelin secretion in this study [55]. These results indicate that although the primary source of ghrelin is the gastric mucosa, small intestinal nutrient exposure is sufficient to decrease postprandial ghrelin levels.

The sweeteners OFS and sucralose were not able to affect plasma ghrelin levels or gastric or duodenal ghrelin content in vivo. These findings, together with the observation that the effect of d-glucose on ghrelin levels is not dependent on signaling through α-gustducin, would argue against a role for α-gustducin mediated sweet taste receptor signaling as glucose sensor of the X/A cell. Also in a dose-escalation study from 0 to 55 g daily of OFS, no significant effects were observed on plasma ghrelin levels [56]. Artificial sweeteners did also not elicit differences in plasma ghrelin levels in healthy subjects in previous studies [57,58]. Furthermore, results comparing equicaloric doses of glucose and fructose observed that the decrease in ghrelin levels after fructose administration, which is sweeter than glucose, was less pronounced [59] or equal [60] to the effect of glucose. These results suggest that the effect of glucose and fructose is not determined by their sweetness.

In fact, a similar discrepancy has been found for the effect of sucralose on GLP-1 release in in vitro and in vivo studies. In enteroendocrine cell lines sucralose stimulates GLP-1 release via the sweet taste receptor [28,61] whereas in vivo studies in humans and rodents fail to demonstrate an effect of sucralose on GLP-1 release [57,62]. The regulatory interface of the GI tract is more complex than the physiological processes mimicked in in vitro experiments and is modulated by multiple homeostatic and non-homeostatic factors. This complexity may explain the discrepancy between in vitro and in vivo findings.

## 5. Conclusions

In conclusion, sensing of d-glucose by the ghrelin cell is polarized, occurs at the luminal side of the duodenum and may overrule gastric glucose sensing. Furthermore, α-gustducin-mediated sweet taste receptor signaling does not play a physiological role in the sensing of carbohydrates and sweeteners by the ghrelin cell since; (1) the effects of d-glucose and sweeteners in the ghrelinoma cell line are not blocked by the sweet taste receptor antagonist gurmarin; (2) d-glucose and the sweeteners affect ghrelin release in gastric segments which do not express one of the subytpes (TAS1R2) of the sweet taste receptor; (3) the effects are not reduced in α-gust^−/−^ mice and (4) the sweeteners OFS and sucralose were not able to elicit the same responses on ghrelin secretion as d-glucose in vivo.

We were unable to show a role for SGLT1 or GLUT2 as glucose sensor of the ghrelin cell and prior data on the involvement of the K_ATP_ channel are inconclusive. Therefore, the role of different G-proteins and the functional role of a TAS1R3 homodimer or K_ATP_ channels as glucose sensors of the ghrelin cell warrant further investigation.
